# Jejum Intermitente no Controle da Hipertensão: Temos Evidências Suficientes?

**DOI:** 10.36660/abc.20230607

**Published:** 2024-03-14

**Authors:** Adriana Camargo Oliveira, Natália de Filippo Mariosa, Marcos Pereira Caetano, Mikaelle Costa Correia

**Affiliations:** 1 Universidade Federal de Goiás Faculdade de Medicina Goiânia GO Brasil Programa de Pós-graduação em Ciências da Saúde, Faculdade de Medicina, Universidade Federal de Goiás (UFG), Goiânia, GO – Brasil; 2 Universidade Federal de Goiás Hospital das Clínicas Liga de Hipertensão Arterial Goiânia GO Brasil Liga de Hipertensão Arterial, Hospital das Clínicas da UFG, Goiânia, GO – Brasil; 3 Universidade Federal de Goiás Faculdade de Farmácia Goiânia GO Brasil Faculdade de Farmácia, Universidade Federal de Goiás UFG, Goiânia, GO – Brasil

**Keywords:** Hipertensão, Jejum, Pressão Arterial, Rigidez Arterial

**Prezado Editor,**

O estudo publicado por Demirci et al.,^1^ traz uma discussão interessante e atual ao avaliar o efeito do jejum intermitente (JI) na pressão arterial, no sistema renina angiotensina (SRA) e no sistema nervoso autônomo (SNA) em pacientes hipertensos através da monitorização ambulatorial da pressão arterial de 24 horas (MAPA), eletrocardiograma Holter e níveis séricos de Ang-I e Ang-II e atividade da ECA antes e após o JI. Entre os achados, destaca-se a redução notável da pressão arterial nos pacientes submetidos ao JI, além da diminuição na expressão da Ang-II e ECA nesse mesmo grupo. Adicionalmente, a diminuição dos níveis de Ang-II foi identificada como um fator preditivo para a melhora da pressão arterial após o JI.

Considerando os resultados apresentados, gostaríamos de discutir sobre alguns pontos que consideramos conflitantes em relação a população estudada. Os participantes apresentavam baixo risco cardiovascular, eram hipertensos controlados e não tinham comorbidades, no entanto, nos resultados de MAPA observamos um grupo com pressão acima dos valores normais, o que poderia sugerir uma hipertensão mascarada não identificada previamente. De forma complementar, os autores não consideram possíveis mudanças no estilo de vida, o que poderia introduzir um viés nos resultados obtidos e estas questões devem ser consideradas na interpretação dos achados e desenho do estudo.

Partindo da ideia de que o Jejum Ramadã (JR) é considerado uma forma de JI até que ponto essa análise seria válida a população brasileira? O JR é um protocolo bem restrito e padronizado da cultura muçulmana e parece não se enquadrar as formas de JI praticadas pelos brasileiros, seja para redução de medidas ou melhora da saúde.^2 , 3^ Acreditamos que a prática do jejum de Ramadã é uma maneira severa de controle da pressão arterial, principalmente neste estudo cuja população supostamente já está controlada e, além de tudo, teria pouca adesão se aplicada à sociedade.

Por fim, para contribuir com os autores, indicamos a realização de estudos de rigidez arterial pela medida da velocidade da onda de pulso (VOP) e índice de aumento (Aix). Essa avaliação não invasiva tem se destacado como um forte preditor de eventos cardiovasculares^4^ e pode complementar as análises de MAPA e Holter nos próximos estudos do grupo. Estudos publicados até o momento são escassos e os resultados conflitantes. Uma coorte de 71 pacientes, avaliou os efeitos do JR nos parâmetros de envelhecimento arterial, AIx e VOP em pacientes hipertensos com e sem doença renal crônica (DRC)^5^ Concluiu que o JR está associado a um melhor controle da pressão arterial periférica e central em pacientes hipertensos com e sem DRC e que também está associada à melhora da complacência arterial (diminuição do AIx) em hipertensos sem DRC.^5^ Portanto, ressaltamos que a avaliação da rigidez arterial pode ser realizada de forma não invasiva pela medida da VOP como uma ferramenta útil na avaliação de estratificação e risco para eventos cardiovasculares podendo ser considerada pelos autores Demirci et al.^1^
