# Supplementary material for: Jejum Intermitente no Controle da Hipertensão: Temos Evidências Suficientes?
Source: Arq Bras Cardiol. 2024 Mar 14;121(3):e20230607. [Article in Portuguese] doi: 10.36660/abc.20230607 (PMC12092017; doi:10.36660/abc.20230607)
Supplement: Translation

# Translation: Intermittent Fasting in the Control of Hypertension: Do We Have Enough Evidence?

**Authors:** Adriana Camargo Oliveira, Natália de Filippo Mariosa, Marcos Pereira Caetano, Mikaelle Costa Correia

**Affiliations:** 1 Goiânia GO Brazil Programa de Pós-graduação em Ciências da Saúde, Faculdade de Medicina, Universidade Federal de Goiás (UFG), Goiânia, GO – Brazil; 2 Goiânia GO Brazil Liga de Hipertensão Arterial, Hospital das Clínicas da UFG, Goiânia, GO – Brazil; 3 Goiânia GO Brazil Faculdade de Farmácia, Universidade Federal de Goiás UFG, Goiânia, GO – Brazil

**Keywords:** Hypertension, Fasting, Blood Pressure, Arterial Stiffness

**Dear Editor,**

The study published by Demirci et al.^1^brings up an interesting and current discussion by evaluating the effect of intermittent fasting (IF) on blood pressure, the renin-angiotensin system (RAS), and the autonomic nervous system (ANS) in hypertensive patients using 24-hour ambulatory blood pressure monitoring (ABPM), Holter electrocardiogram, serum levels of Ang-I and Ang-II and ACE activity before and after IF. Among the findings was a notable reduction in blood pressure in patients undergoing IF, as well as a decreased expression of Ang-II and ACE in this same group. In addition, the reduction in Ang-II levels was identified as a predictive factor for blood pressure improvement after IF.

Considering the results presented, we would like to discuss some points that we consider to be conflicting about the population. The participants had low cardiovascular risk and were controlled hypertension and had no comorbidities, however, in the MAP results, we observed a group with pressure above normal values, which could suggest normal values and masked hypertension not previously identified. In addition, the authors did not consider the possible lifestyle changes, which could introduce a bias in the results obtained. These issues should be considered when interpreting the findings and study design.

Considering the results presented, we would like to discuss some points that we consider conflicting in relation to the studied population. The participants had low cardiovascular risk, were controlled hypertensive, and had no comorbidities; however, in the ABPM results, we observed a group with pressure above normal values, which could suggest previously unidentified masked hypertension. Additionally, the authors do not consider possible changes in lifestyle, which could introduce a bias in the results obtained, and these issues must be considered when interpreting the findings and designing the study.

Based on the idea that Ramadan Fasting (RF) is considered, how valid this analysis would be for the Brazilian population? RF is a standardized protocol of Muslim culture and does not seem to fit in with the forms of IF practiced by Brazilians, either to reduce blood pressure or health improvement.^2 , 3^We believe that fasting during Ramadan is a severe way of controlling blood pressure, especially in this study, whose population is supposedly already under control and, moreover, would have little adherence if applied to society.

Finally, to help the authors, we recommend carrying out studies of arterial stiffness by measuring pulse wave velocity (PWV) and augmentation index (Aix). This non-invasive assessment has stood out as a strong predictor of cardiovascular events^4^and can complement the analysis in the group’s forthcoming studies. Studies published to date are scarce, and the results are conflicting. A cohort of 71 evaluated the effects of JR on arterial aging parameters, AIx, and OPV in hypertensive patients with and without chronic kidney disease (CKD).^5^It concluded that RF is associated with better control of peripheral and central blood pressure in hypertensive patients with and without CKD and it is associated with improved arterial compliance (decreased AIx) in hypertensive patients without CKD.^5^Therefore, we emphasize that the assessment of arterial stiffness can be carried out non-invasively by measuring PWV as a useful tool in assessing risk stratification for cardiovascular events and can be considered by the authors Demirci et al.^1^
